# Residual bile flow shapes PPARγ-mediated antifibrotic responses in experimental segmental cholestasis

**DOI:** 10.1038/s41598-026-50023-1

**Published:** 2026-04-25

**Authors:** Josiane O. Gonçalves, Cleiton A. M. Mafra, Isac Castro, Bruno Cogliati, Silvia Y. Bando, Walcy R. Teodoro, Suellen Serafini, Lorenzo F. Ferreira, Carlos A. Moreira-Filho, Uenis Tannuri, Ana C. A. Tannuri

**Affiliations:** 1 Division of Pediatric Surgery, Pediatric Liver Transplant Unit, Pediatric Surgery Research Laboratory (LIM 30), University of São Paulo School of Medicine, Av. Dr. Arnaldo, 455 – Cerqueira César, São Paulo, SP 01246-903 Brazil; 2 Department of Pediatrics, University of São Paulo School of Medicine, São Paulo, SP Brazil; 3 Liver and Gastrointestinal Transplant Division, Department of Gastroenterology, University of São Paulo School of Medicine, São Paulo, SP Brazil; 4 Department of Nephrology, University of São Paulo School of Medicine, São Paulo, SP Brazil; 5 Department of Pathology, School of Veterinary Medicine and Animal Science, University of São Paulo, São Paulo, SP Brazil; 6 Division of Rheumatology, Hospital das Clínicas, University of São Paulo School of Medicine, São Paulo, SP Brazil

**Keywords:** Liver fibrosis, Segmental cholestasis, PPARγ, Pioglitazone, Fibro-inflammatory signaling, Gene regulatory networks, Diseases, Gastroenterology, Medical research

## Abstract

Segmental cholestasis represents a clinically relevant but underexplored cause of progressive liver fibrosis, particularly in pediatric conditions associated with intrahepatic biliary strictures, where segmental obstruction promotes progressive fibro-inflammatory injury that extends into adjacent non-cholestatic parenchyma. The mechanisms determining regional therapeutic responsiveness in segmental cholestasis remain poorly defined. . Here, we investigated the effects of PPARγ agonism in a selective bile duct ligation (sBDL) model that reproduces key features of segmental cholestasis and the propagation of fibro-inflammatory signaling beyond the primary site of injury. Newly weaned Wistar rats underwent sBDL and were treated with the PPARγ agonist pioglitazone or vehicle. Cholestatic (CL) and non-cholestatic (NCL) liver lobes were analyzed at 7, 30, and 60 days post-surgery using histological, immunohistochemical, biochemical, transcriptomic, and gene co-expression network analyses. Therapeutic responsiveness was strongly region dependent. In non-cholestatic lobe (NCL-T), PPARγ activation reduced macrophage recruitment, ductular reaction, and collagen deposition, accompanied by downregulated inflammatory and TGF-β–related signaling pathways. Gene co-expression network analysis revealed that PPARγ activation showed a hierarchical gene network topology and functional modularity in treated non-cholestatic lobes, preventing the network collapse observed in vehicle-treated animals. In contrast, the cholestatic lobe (CL-T) showed limited transcriptional responsiveness and remained largely refractory to therapeutic modulation, consistent with a stabilized fibro-inflammatory microenvironment. These findings demonstrate that injury heterogeneity and partial preservation of bile flow critically determine of therapeutic responsiveness, supporting PPARγ activation as a preventive antifibrotic strategy to limit fibro-inflammatory propagation in vulnerable hepatic regions during segmental cholestasis.

**Supplementary Information:**

The online version contains supplementary material available at 10.1038/s41598-026-50023-1.

## Introduction

Obstructive cholestasis is a common feature of several pediatric liver diseases, including biliary atresia, cholelithiasis, sclerosing cholangitis, and intrahepatic biliary strictures, and represents a major cause of chronic liver disease and transplantation in children.^1–3^ The interruption of bile flow induces hepatocellular injury and activates innate immune signaling through the release of damage-associated molecular patterns (DAMPs), leading to ductular reaction and recruitment of inflammatory cells.^4^ Persistent cholestatic injury disrupts normal tissue repair mechanisms, resulting in excessive extracellular matrix deposition, progressive fibrosis, and ultimately biliary cirrhosis^5^.

When biliary obstruction is restricted to specific segments of the intrahepatic biliary tree, as observed in peripheral or segmental strictures, fibrogenesis is not limited to the cholestatic segment but propagates into adjacent lobes that retain preserved bile drainage.^6,7^ Experimental and clinical evidence indicates that, during segmental cholestasis, reactive cholangiocytes act as central drivers of this process by releasing paracrine and autocrine signals that shape the local inflammatory microenvironment. These signals modulate crosstalk with immune cells, portal fibroblasts, and hepatic stellate cells, thereby sustatining inflammatory activation, myofibroblastic transdifferentiation, and extracellular matrix accumulation in non-cholestatic parenchyma.^8,9^ This phenomenon contribute to progressive cirrhosis despite partial preservation of bile flow, a scenario particularly detrimental in the immature liver.

In the developing liver, cellular populations involved in fibrogenesis exhibit age-dependent biological behavior. Hepatic stellate cells (HSCs), the principal fibrogenic cells in the liver, display enhanced plasticity and heightened responsiveness to injury-related signals during early developmental stages.^10–13^ Upon Activation, HSCs transdifferentiate into collagen-producing myofibroblasts, a key event in hepatic fibrogenesis and are strongly influenced by the inflammatory and biliary microenvironment created during cholestatic injury.^14–16^ These developmental characteristics may influence both the extend and spatial distribution of fibrotic remodeling following biliary obstruction.^15^

Segmental biliary strictures are particularly relevant in pediatric hepatology. They occur in approximately 20–30% of pediatric liver transplant recipients and represent one of the most frequent post-transplant complications.^2,17,18^ While extrahepatic strictures often respond to interventional procedures,^19^ intrahepatic strictures, typically peripheral and segmental, remain a major clinical challenge in children. These lesions are frequently associated with persistent cholestasis, progressive secondary fibrosis, and an increased risk of graft dysfunction, or retransplantation during childhood or adolescence.^20^ Despite their clinical impact, therapeutic strategies capable of modulating the fibrogenic response triggered by segmental cholestasis in pediatric patients are currently lacking.

Experimental models of biliary obstruction have been used to investigate mechanisms of cholestatic liver injury. However, the classical common bile duct ligation (BDL) model induces diffuse cholestasis and does not mimic the spatial heterogeneity characteristic of segmental biliary strictures observed in pediatric disease. To overcome this limitation, other studies and we have employed the selective bile duct ligation (sBDL) model in weaned rats, which reproduces segmental cholestasis and the propagation of fibro-inflammatory signaling into adjacent non-cholestatic lobes (NCL).^21–23^ Importantly, the use of young animals allows the investigation of fibrogenic responses within a developmental context that more closely reflects pediatric liver disease, in which hepatic repair mechanisms and stellate cell activation occur within an immature and dynamically envolving hepatic microenvironment.^24^ Thus, sBDL represents a particularly suitable model to investigating antifibrotic strategies targeting segmental cholestasis, a condition that remains poorly studied.

Peroxisome proliferator–activated receptor gamma (PPARγ) agonists have emerged as potential modulators of inflammatory and profibrotic pathways in several experimental models.^25–29^ Nevertheless, their antifibrotic efficacy appears to depend on the type and severity of hepatic injury. Pioglitazone attenuates fibrosis in hepatotoxic models, such as carbon tetrachloride (CCl₄) induced injury, but shows limited or variable efficacy in models of diffuse cholestasis induced by BDL.^30^ Beneficial effects have been reported primarily when treatment is initiated preventively, before the onset of cholestatic injury.^31,32^ It remains unclear whether PPARγ activation is able to modulate fibrogenesis in segmental cholestatic lesions, particularly in the immature liver.

In this study, we investigated whether pharmacological activation of PPARγ can modulate fibrogenesis induced by segmental cholestasis and limit the propagation of fibro-inflammatory signaling into hepatic regions with preserved bile flow. Using the sBDL model in young rats, we examined the effects of PPARγ agonist treatment on segmental cholestasis–induced fibrosis and characterized the major pathways modulated by this treatment.

## Methods

### Ethical approval

All procedures were approved by the Animal Ethics Committee (CEUA) of the University of São Paulo Medical School (protocol 489/13) and conducted in accordance with the ARRIVE guidelines and the Brazilian Guide for the Care and Use of Laboratory Animals (CONCEA). Every effort was made to minimize animal suffering.

### Animal care

Sixty-two newly weaned male and female Wistar rats (*Rattus norvegicus albinus, Rodentia, Mammalia*), obtained from the Animal Facility of the Institute of Biomedical Sciences, University of São Paulo (ICB-USP), aged 21–23 days and weighing 60-80 g, were used. Recently weaned animals were selected to model a developmental stage in which the liver is still undergoing postnatal maturation, allowing investigation of fibrogenic responses within a biological context more closely resembling early pediatric liver disease.^33^ Animals were housed in same-sex pairs in polyethylene cages (49 x 34 x 16 cm) with sawdust bedding under controlled environmental conditions (22 ± 2 ºC, 55 ± 10% relative humidity, 12-hour light/dark cycle). Standard commercial chow (Nuvilab CR-1, Quimtia SA) and water were provided *ad libitum*.

### Experimental design

Rats were randomly allocated to experimental groups using a simple randomization strategy and divided into three groups: selective bile duct ligation (sBDL), selective bile duct ligation treated with PPARγ agonist (sBDL-T), and sham surgery. Animals were anesthetized with ketamine hydrochloride (100 mg/kg) and xylazine hydrochloride (30 mg/kg). Adequate anesthesia was confirmed by absence of the eyelid reflex, no response to painful stimuli, and regular respiration patterns.

A supra-umbilical midline laparotomy was performed to expose the liver. After the organ was exposed, the animals underwent selective bile duct ligation, as previously described.^13^ In the sBDL and sBDL-T groups, bile ducts draining the median, left lateral, and caudate lobes (~76% of liver mass) were ligated and sectioned (cholestatic lobe – CL or CL-T), while the right lateral lobe remained intact (non-cholestatic lobe – NCL or NCL-T). Sham-operated animals underwent the same laparotomy and bile duct dissection, without ligation or transection, replicating the surgical conditions of sBDL without inducing biliary obstruction. All procedures were performed using microsurgical instruments and a stereomicroscope (DF Vasconcelos®, Valença, Brazil) to preserve hepatic artery and portal vein branches. Postoperative analgesia was provided with intramuscular tramadol hydrochloride (15 mg/kg) twice daily for 7 consecutive days. Animals were weighed, anesthetized, and blood was collected via cardiac puncture prior to euthanasia by isoflurane overdose at 7, 30, and 60 days post-surgery. Sham-operated animals were evaluated at the same time points, and animals from each experimental group were compared with age-matched sham controls (n = 6 rats/group).

### Administration of the PPAR-gamma agonist

Animals subjected to sBDL were blindly randomized to receive pioglitazone hydrochloride (10 mg/kg/day) suspended in 0.5% CMC-Na (sBDL-T) or vehicle (0.5% CMC-Na; sBDL). The dose of pioglitazone was selected based on previous experimental studies demonstrating antifibrotic and anti-inflammatory effects of PPARγ activation in rodent models of liver injury.^34–36^ Treatments were administered once daily by oral gavage starting on postoperative day 3 and maintained until euthanasia. The choice of postoperative day 3 for treatment initiation was based on prior evidence showing that key pro-fibrotic mediators, including TGF-β1, pro-inflammatory cytokines, and early hepatic stellate cell activation, are already upregulated at this time point following biliary obstruction, marking the onset of active fibrogenesis.^37^ Initiating therapy after this activation phase allowed evaluation of PPARγ agonism as a therapeutic intervention, rather than as a prophylactic strategy.

### Tissue and serum collection

The liver was removed, flushed with 0.9% saline, and weighed. The CL-T/CL and NCL-T/NCL lobes were separated and divided into three portions: (i) fixed in 10% buffered formalin for 24 h at room temperature for histological and immunohistochemical analysis; (ii) snap-frozen in liquid nitrogen (-180 °C) for hydroxyproline quantification; and (iii) stored in RNAlater™ (Thermo Fisher Scientific, Waltham, MA) at -80 °C for molecular analyses. Blood samples were centrifuged at 4,000 rpm for 10 min at 4 °C to obtain serum for biochemical analysis, including aspartate aminotransferase (AST), alanine aminotransferase (ALT), gamma-glutamyl transferase (GGT), alkaline phosphatase, and total bilirubin.

### Hydroxyproline content

Frozen liver tissue (50–100 mg) was hydrolyzed in 6 N HCl at 95 °C for 24 h. Hydroxyproline concentration was determined colorimetrically and expressed as µg hydroxyproline per mg tissue.^38^

### Histology and immunohistochemistry

Paraffin-embedded liver samples were sectioned at 4 µm and mounted on silanized slides (Star Frost, Waldemar Knittel, Germany). Sections were deparaffinized in xylene and rehydrated through graded ethanol series. Histological staining was performed with hematoxylin and eosin (H&E) and Picro-Sirius Red for collagen visualization. H&E slides were evaluated at 400× for portal and periportal inflammatory infiltrates and hepatocellular injury. Inflammatory infiltrates were scored: 0 (absent), 1 (mild), 2 (moderate), 3 (severe). Collagen content was assessed in 10 randomly selected 200× fields, expressed as percentage of collagen relative to portal area (µm2).

Immunohistochemical staining was performed for cytokeratin-19 (CK-19, bile ducts), CD68 (macrophages/Kupffer cells), α-smooth muscle actin (α-SMA, activated myofibroblasts), Ki-67 (proliferating cells), and cleaved caspase-3 (apoptotic cells). Antigen retrieval was done using a Riptide pressure chamber (Celerus Diagnostics, USA) at 125 °C and 20 psi for 2 min in 0.05% citraconic anhydride (pH 7.4). Endogenous peroxidase was blocked with 3% H₂O₂, followed by TBST buffer washing (pH 7.4). Non-specific binding was blocked using Novolink™ protein block (Leica Biosystems, UK). The slides were incubated overnight at 4 °C with the following primary antibodies diluted in 1% BSA buffer: anti-CK-19 (PA5-87389, 1:1500, Thermo Fisher Scientific, USA), anti-CD68 (PA5-81594, 1:1500, Invitrogen, USA), anti-α-SMA (1A4, SC32251, 1:300, Santa Cruz Biotechnology, USA), anti-Ki-67 (SP6, M3064, 1:1000, Spring Bioscience, USA), and cleaved anti-caspase-3 (9661, 1:3000, Cell Signaling Technology, USA). Detection was performed using Novolink™ HRP polymer system (Leica Biosystems, UK) according to the manufacturer’s instructions.

Quantification was performed on 10 random 200× fields for CK-19, CD68, and α-SMA. Ki-67 was assessed in 1,000 hepatocytes per sample, and cleaved caspase-3 in 20 random 400× fields. Areas positive for CK-19, CD68, and α-SMA were expressed as percentage of the total analyzed area (µm^2^); Ki-67 index as percentage of positive nuclei among 1000 cells relative to the total analyzed area (mm^2^); apoptotic index as cleaved caspase-3-positive cells per mm^2^. Photomicrographs were acquired with Axio Lab. A1 microscope (Carl Zeiss, Germany) using AxioVs40 software (v4.8.2.0), and analysis was performed using Image-Pro Plus (v4.5.0.29, Media Cybernetics, USA). All histological and immunohistochemical analyses were performed by an experienced veterinary pathologist blinded to experimental groups.

### qRT-PCR array and gene expression analysis

#### RNA isolation and qRT-PCR array

Total RNA was extracted from liver samples using the RNeasy kit (Qiagen, San Diego, CA) according to the manufacturer’s instructions. RNA quantity and purity were assessed using a Biophotometer spectrophotometer (Eppendorf AG, Germany), and integrity was verified on 1.2% agarose gels. cDNA synthesis was performed using the RT2 First Strand Kit (Qiagen, San Diego, CA), following the manufacturer’s protocol. RNA and cDNA quality were further validated using the RT2 RNA QC PCR Array (PARN-999Z, Qiagen, San Diego, CA).

Gene expression was analyzed using the RT2 Profiler PCR Array for rat fibrosis (PARN-120Z, Qiagen, Germany) in Rotor-Disc 100 format, following OEM recommendations. Reactions were run on a Rotor-Gene Q 5plex HRM thermocycler (Qiagen, Germany), and cycle threshold (Ct) values were obtained using Rotor-Gene Q Pure Detection software (v2.0.3, Qiagen, Germany). Raw Ct values were uploaded to the Qiagen GeneGlobe Data Analysis Center (www.qiagen.com/geneglobe). The array includes five candidate housekeeping genes (Actb, B2m, Hprt1, Ldha, and Rplp1), and normalization was performed using the geometric mean of the most stable genes identified by the GeneGlobe Data Analysis Center, namely Hprt1 and Rplp1. Fold changes were calculated on a log₂ scale relative to the sBDL group. Genes with 2.0-fold change were considered differentially expressed.

Differentially expressed genes were visualized using volcano plots, in which the x-axis represents the log2 fold change and the y-axis represents the −log10 of the p-value. Genes were considered significantly differentially expressed when |log2FC| ≥ 1 and −log10(p-value) ≥ 1.3. Genes were further classified according to their transcriptional response to treatment. Effective normalization was defined as a residual log2 fold change <0.25 in treated with PPARγ agonist animals relative to sham controls. Incomplete normalization was assigned when gene expression remained between 0.25 and 1.0 log2 fold change from sham levels, despite significant modulation compared with vehicle-treated disease. Over-suppression was defined as gene expression levels in treated with PPARγ agonist animals falling below sham baseline levels (log2 fold change ≤ −1).

### Validation of qRT-PCR array

Key dysregulated genes identified from the PCR array were validated using gene-specific primers designed and evaluated in silico with NCBI Primer-BLAST, based on *Rattus norvegicus* sequences from GenBank. Candidate reference genes (*HPRT, B2M, GAPDH*, and *YWHAZ*) were assessed for expression stability across all experimental groups using the RefFinder tool,^39^ which integrates geNorm, NormFinder, BestKeeper. RefFinder generated a comprehensive stability ranking (Supplementary Material 1), and *HPRT* was identified as the most stable reference gene. The sequences of all primers and their respective amplicon sizes are listed in Table 1.

**Table 1:** Primer sequences and in silico specificity analysis for *Rattus norvegicus* target genes used in qRT-PCR experiments for validation of the qRT-PCR array.

**Gene**	**Variant/ Accession**	**Primer**	**Sequence (5’→3’)**	**Expected amplicon (bp)**	**Blast top hit**	**Identity**	**In silico specificity**
*Acta2*	NM_031004.2	Forward	GGATCAGCGCCTTCAGTTCT	108	*Acta2 (Rattus norvegicus)*	100%	Single specific product predicted
		Reverse	CAGGGCTAGAAGGGTAGCAC				
*Ccl3*	NM_013025.2	Forward	CCAAGTAGCCACATCCAGGG	142	*Ccl3 (Rattus norvegicus)*	100%	Single specific product predicted
		Reverse	ATGTGCCCTGAGGTCTTTCAG				
*Col1a2*	NM_053356.2	Forward	GAAATGGCAACTCAGCTCGC	87	*Col1a2 (Rattus norvegicus)*	100%	Single specific product predicted
		Reverse	CGCAATGCTGTTCTTGCAGT				
*Lox*	NM_001414003.1	Forward	CAGGCACCGACCTGGATATG	145	*Lox (Rattus norvegicus)*	100%	Single specific product predicted
		Reverse	CAGGCAGTTTTCTTCCGCAG				
*Mmp2*	NM_031054.2	Forward	GGTGGCAATGGAGATGGACA	130	*Mmp2 (Rattus norvegicus)*	100%	Single specific product predicted
		Reverse	CCCGGTCATAATCCTCGGTG				
*Nfkb1*	NM_001276711.2	Forward	AAGACAGCACATAGATGAGCTCCG	116	*Nfkb1 (Rattus norvegicus)*	100%	Specific for Nfkb1 mRNA (NM_001276711.2 and NM_001415012.1)
		Reverse	ATAGCAGTGGGCCATCTCCA				
*Stat6*	NM_001044250.1	Forward	CGCTCATAAGCCGTCTGGAT	119	*Stat6 (Rattus norvegicus)*	100%	Single specific product predicted
		Reverse	GAATCGAACCCCAGCCTGAA				
*Tgfb3*	NM_013174.2	Forward	CCCGGCAGAACCTGTTTAGAT	154	*Tgfb3 (Rattus norvegicus)*	100%	Single specific product predicted
		Reverse	AAGACTGAGGCTTGGCAAGAG				
*Tgif1*	NM_001015020.1	Forward	CCAGTGTCTCCCAAACCTCC	98	*Tgif1 (Rattus norvegicus)*	100%	Single specific product predicted
		Reverse	GAGAAAGGCCCGTCCTTCAA				
*Hprt1*	NM_012583.2	Forward	CTTCCTCCTCAGACCGCTTT	79	*Hprt1 (*Rattus norvegicus*)*	100%	Single specific product predicted
		Reverse	TCACTAATCACGACGCTGGG				

Variant / Accession: GenBank or RefSeq identifier of the specific mRNA isoform for which primers were designed.

Expected amplicon (bp): Predicted PCR product size in base pairs.

In silico specificity: Primer specificity was evaluated using NCBI Primer-BLAST, confirming exclusive binding to the intended Rattus norvegicus target mRNA and absence of significant off-target amplification.

qRT-PCR reactions were performed in 20 μL volumes containing 100 ng cDNA, 0.4 μL forward and reverse primers (10 μM each), and 10 μL Platinum SYBR Green qRT-PCR SuperMix-UDG (Invitrogen). Thermal cycling: 95 °C for 5 min; 40 cycles of 95 °C for 20 s, 60–62 °C for 30 s, and 72 °C for 30 s, with fluorescence detected at 72 °C. Each sample was analyzed in triplicate with no-template controls (NTCs). Specificity was confirmed via melting curve analysis. Relative expression was calculated using the 2^-ΔΔCt method.^40^

### Gene Co-expression network analysis

To explore gene interactions and identify hub genes in fibrogenesis, gene co-expression networks were constructed from expression data. The gene co-expression networks were constructed by Pearson correlation analysis for each group CL, CL-T, NCL, and NCL-T. Gene-gene correlation cutoff value ≥ |0.70| was adopted for all groups. Networks visualizations and analyses was performed in Cytoscape v3.8.2.^41^ Node degree was used to identify hub genes and subsequently their first neighbors genes.

### Functional analysis for module genes in the gene co-expression networks

A subset of genes from each gene co-expression networks was used for enrichment analysis. The gene list was composed by the module genes from networks presenting hierarchical topology or the subset of the genes encompassing the hub gene and its first neighbor genes from disorganized networks. Enrichment analysis was conducted using the Enrichr web-based platform (https://maayanlab.cloud/Enrichr/),^42,43^ focusing on the Reactome pathway database. Terms were considered significant at p < 0.0001 and filtered by clear pathophysiological relevance to the disease context. Representative pathways were subsequently selected for network visualization.

### Statistical analysis

GraphPad Prism v9.5.1 was used for statistical analyses. Normality was assessed with the Shapiro-Wilk test. Differences among groups were analyzed using one-way ANOVA with Tukey post hoc or Kruskal-Wallis with Dunn post hoc test and Benjamini-Hochberg correction.

Gene expression analysis was performed using the RT2 Profiler PCR Array Data Analysis software. Differential expression was determined using Student’s *t*-tests comparing PPAR-γ agonist-treated groups (CL-T and NCL-T) with their respective vehicle-treated controls (CL and NCL), followed by Benjamini–Hochberg correction. A two-tailed *p* value < 0.05 was considered statistically significant.

## Results

### General findings

**Fig. 1:**
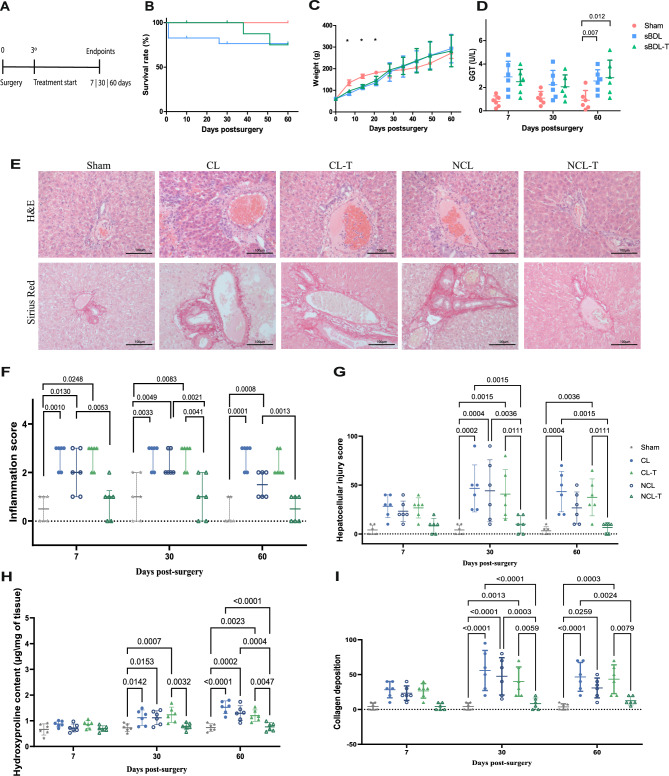
General experimental assessments and liver injury in the sBDL model. (**A**) Experimental design illustrating selective bile duct ligation (sBDL), treatment allocation, and time points of analysis. (**B**) Survival analysis of rats in the sham, sBDL, and sBDL-T groups. The blue line represents the sham group, the green line represents animals subjected to sBDL, and the pink line represents sBDL-T animals treated with pioglitazone. Kaplan–Meier survival curves were compared using the log-rank test (p = 0.0896). (**C**) Body weight variation during the experimental period. (**D**) Serum gamma-glutamyltransferase (GGT) levels at the indicated time points. (**E**) Representative histological sections from Sham, cholestatic lobe (CL), non-cholestatic lobe (NCL), pioglitazone-treated cholestatic lobe (CL-T), and pioglitazone-treated non-cholestatic lobe (NCL-T). Hematoxylin and eosin (H&E) staining illustrates morphological alterations, while Sirius Red staining highlights collagen deposition in portal and periportal regions. Images correspond to liver sections obtained 60 days after surgery (original magnification, ×200). (F–I) Quantitative histological and biochemical analyses: inflammatory infiltrate (**F**), hepatocellular injury score (**G**), hepatic hydroxyproline content (**H**), and collagen deposition quantified by Sirius Red staining (**I**). Data are expressed as mean ± SEM. Statistical analysis was performed using one-way ANOVA followed by appropriate post hoc tests.

Selective bile duct ligation (sBDL) resulted in a mortality rate of 12.9%, occurring predominantly within the first 24 h after surgery (n = 5), and isolated events on postoperative days (POD) 26, 38, and 51. No mortality was observed in sham-operated animals (p = 0.0896). Rats subjected to sBDL, regardless of treatment, exhibited a transient reduction in body weight during the first twenty postoperative days, followed by progressive recovery thereafter. Consistent with sustained cholestatic injury, serum γ-glutamyl transferase (GGT) levels remained significantly elevated in both vehicle-treated (sBDL) and pioglitazone-treated (sBDL-T) groups at 60 days post-surgery (p = 0.007 and p = 0.012, respectively), confirming effective and persistent model induction (Fig. 1). No significant differences were observed among experimental groups in serum ALT, AST, alkaline phosphatase, or total bilirubin levels at the evaluated time points (data not shown).

### PPAR-γ agonism protects the non-cholestatic lobe from hepatocellular injury and fibrosis

PPAR-γ activation produced a clear lobe-specific protective effect in segmental cholestasis. In the non-cholestatic lobe of PPAR-γ agonist-treated rats (NCL-T), portal and periportal inflammation and hepatocellular injury were markedly attenuated compared with vehicle-treated NCL and with both cholestatic lobes (CL and CL-T) (p < 0.05). Despite these protective effects in NCL-T, the cholestatic lobe of PPAR-γ agonist-treated animals (CL-T) did not exhibit significant histological improvement relative to CL (Fig. 1E-G).

Consistent with these findings, vehicle-treated animals exhibited marked collagen deposition in portal areas and elevated hydroxyproline levels in both CL and NCL. PPAR-γ agonism prevented fibrosis progression exclusively in NCL-T, while CL-T showed no significant differences from vehicle-treated CL, indicating that the protective effects of PPAR-γ activation are spatially restricted to areas not directly exposed to obstructive injury (Fig. 1H-I).

### PPAR-γ agonism reduces macrophage recruitment, ductular reaction, and myofibroblast accumulation in the non-cholestatic lobe

**Fig. 2:**
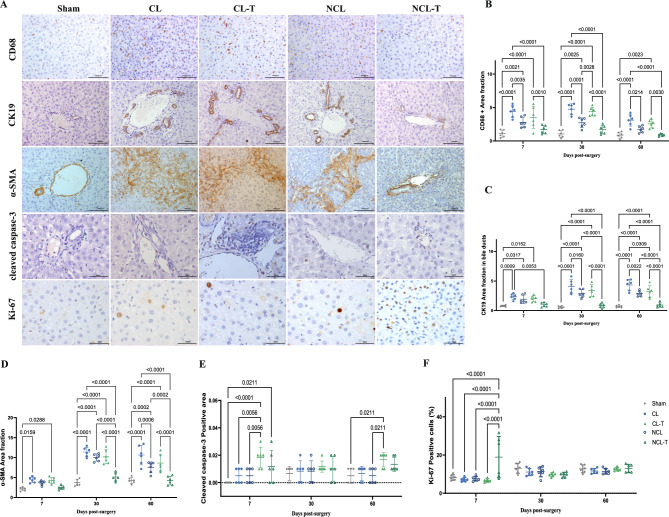
Immunohistochemical characterization of inflammation, ductular reaction, fibrogenesis, apoptosis, and proliferation. (**A**) Representative immunohistochemical staining in Sham, CL, NCL, CL-T, and NCL-T. CD68 immunostaining (brown) identifies macrophages; CK19 immunostaining (brown) marks ductular reaction; and α-SMA immunostaining (brown) identifies activated myofibroblasts 30 days after surgery (original magnification, ×200). Cleaved caspase-3 immunostaining (brown) was used to evaluate apoptosis, and Ki-67 immunostaining (brown) was used to assess cell proliferation 7 days after surgery (original magnification, ×400). (B–F) Quantitative analysis of immunostaining: CD68-positive macrophages (**B**), CK19-positive ductular structures (**C**), α-SMA-positive myofibroblasts (**D**), apoptosis index determined by cleaved caspase-3 (**E**), and proliferation index assessed by Ki-67 (**F**). Data are presented as mean ± SEM. Statistical analyses were performed using Kruskal–Wallis test followed by Dunn’s post hoc test with Benjamini–Hochberg correction, or one-way ANOVA followed by Tukey’s post hoc test, as appropriate (p < 0.05).

CD68 immunostaining revealed marked macrophage accumulation in both CL and NCL of vehicle-treated rats at 7 and 30 days. Pioglitazone prevented macrophage infiltration in NCL-T, but macrophage accumulation persisted at high levels in CL-T. At 60 days, vehicle-treated NCL exhibited a spontaneous reduction in CD68⁺ cells. These spatially distinct patterns were mirrored at the transcriptional level. In vehicle-treated animals, *CCR2* and associated chemokines (*Ccl3*), inflammatory mediators (*Il1b, Nfkb1, Stat6*) were upregulated in both CL and NCL. Pioglitazone downregulated this axis in NCL-T. In CL-T, inflammatory transcripts (*Il1b, Nfkb1, Tnf, Ccl3, Il10, Cxcr4*) were also reduced, though this was not accompanied by decreased CD68⁺ cell numbers (Fig. 2A; 2B).

CK19 immunostaining demonstrated a persistent and marked ductular reaction in CL and CL-T across all time points. In the non-cholestatic lobe, vehicle-treated NCL developed progressive ductular expansion at 30 and 60 days (p < 0.0001) (Fig. 2A; 2C). In contrast, PPAR-γ activation completely prevented this response in NCL-T. The α-SMA⁺ myofibroblasts were abundant in CL and CL-T from 7 to 60 days (p < 0.05), consistent with sustained fibrogenesis in obstructed tissue. In vehicle-treated NCL, α-SMA progressively increased at 30 and 60 days, whereas pioglitazone prevented this accumulation in NCL-T (p < 0.0001) (Fig. 2A; 2D).

At 7 days, cleaved caspase-3 staining indicated increased apoptosis in CL-T versus both CL (p = 0.003) and sham (p < 0.0001), and NCL-T also showed higher apoptotic indices (p = 0.013). No significant differences were detected at 30 days. At 60 days, apoptosis increased again in CL-T compared to sham (p = 0.021) (Fig. 2A; 2E). Ki-67 staining revealed robust early hepatocyte proliferation in NCL-T at 7 days (p < 0.0001), supporting a compensatory regenerative response. No significant differences were observed at 30 or 60 days (Fig. 2A; 2F).

### PPAR-γ agonism remodels the transcriptomic response to segmental cholestasis

**Fig. 3:**
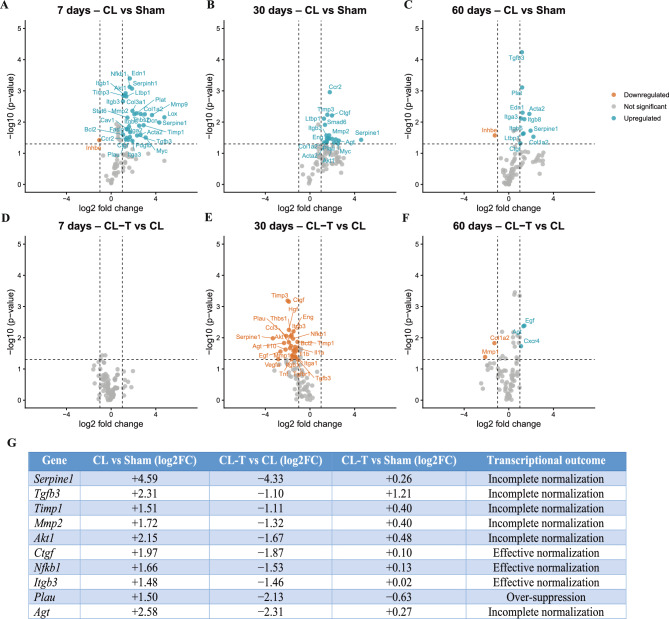
Pioglitazone modulates fibrogenesis-related gene expression in the cholestatic lobe. (**A–C**) Volcano plots showing differentially expressed genes related to fibrogenesis in the cholestatic lobe (CL) of vehicle-treated animals at 7, 30, and 60 days after surgery. (**D**–**F**) Volcano plots of fibrogenesis-related genes in pioglitazone-treated cholestatic lobes (CL-T) at the same time points. The x-axis represents log₂ fold change, and the y-axis represents −log₁₀ (p-value). Colored dots indicate differentially expressed genes (|log_2_ fold change| ≥ 1 and adjusted p < 0.05), including both upregulated and downregulated genes, whereas gray dots represent non-significant genes (t-test with Benjamini–Hochberg correction). (**G**) Table summarizing genes modulated by pioglitazone treatment that were also significantly dysregulated in disease at 30 days, corresponding to the time point of maximal therapeutic effect.

As reported previously,^23^ segmental cholestasis induced robust inflammatory, profibrotic and ECM-remodeling transcriptional signatures in both CL and NCL, confirming effective model induction. In CL, differential gene expression compared with sham animals was most pronounced at 7 and 30-days (Fig. 3A-B) and partially subsided by 60 days (Fig. 3C), consistent with progression from acute injury to a more chronic adaptive phase.

Compared with vehicle-treated animals, PPAR-γ agonism attenuated inflammatory, TGF-β–related, and ECM-remodeling pathways in CL-T, particularly at 30 days (Fig. 3E). This attenuation involved coordinated downregulation of cytokine signaling regulators, components involved in latent TGF-β activation, and genes related to matrix turnover. To systematically characterize treatment-induced transcriptional remodeling, genes dysregulated in disease (CL vs. sham; Fig. 3A-C) were further classified according to their expression profile after pioglitazone treatment (CL-T vs CL; Fig. 3D-F) as showing effective normalization, incomplete normalization, or over-suppression. This gene-level classification is summarized in Fig. 3G. Genes exhibiting partial attenuation of differential expression without full restoration to sham levels were classified as incomplete normalization, whereas genes returning to near-sham expression were considered effectively normalized, and genes suppressed below sham levels were categorized as over-suppressed.

Despite this transcriptional attenuation, structural markers of myofibroblast activation (*Acta2, Col1a2)* remained unchanged in CL-T (Fig. 3E), indicating that PPAR-γ signaling modulates upstream regulatory pathways without reversing established ECM accumulation. By 60 days, CL-T exhibited a late-remodeling transcriptional profile characterized by downregulation of matrix-related genes (*Col1a2, Mmp1*) and modest activation of growth factor signaling (Fig. 3F), consistent with reduced active fibrogenesis and progression toward matrix stabilization.

**Fig. 4:**
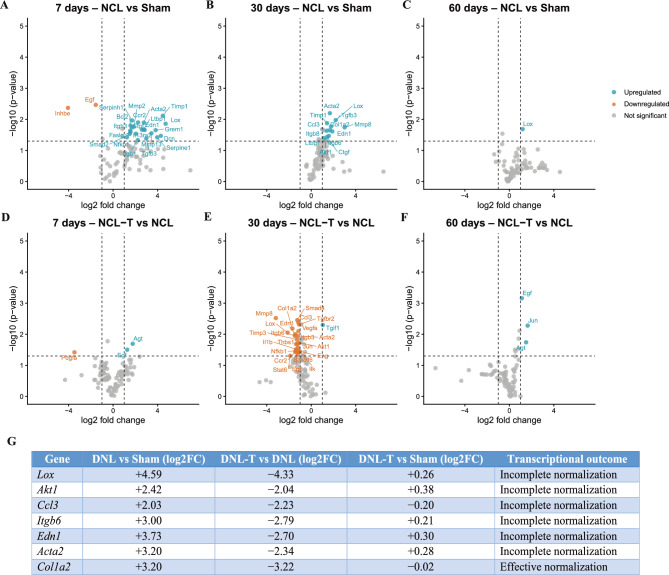
Pioglitazone modulates fibrogenesis-related gene expression in the non-cholestatic lobe. (**A–C**) Volcano plots showing differentially expressed genes related to fibrogenesis in the non-cholestatic lobe (NCL) of vehicle-treated animals at 7, 30, and 60 days after surgery. (**D–F**) Volcano plots of fibrogenesis-related genes in pioglitazone-treated non-cholestatic lobes (NCL-T) at the same time points. The x-axis represents log₂ fold change, and the y-axis represents −log₁₀ (p-value). Colored dots indicate differentially expressed genes (|log_2_ fold change| ≥ 1 and adjusted p < 0.05), including both upregulated and downregulated genes, whereas gray dots represent non-significant genes (t-test with Benjamini–Hochberg correction). (**G**) Table summarizing genes modulated by pioglitazone treatment that were also significantly dysregulated in disease at 30 days, corresponding to the time point of maximal therapeutic effect.

In the absence of treatment, NCL exhibited broad inflammatory and fibrogenic activation despite not being mechanically obstructed, indicating the contribution of extra-ductal autocrine and paracrine signaling during segmental cholestasis. Similar to CL, differential gene expression compared with sham was most evident at 7 and 30 days (Fig. 4A-B) and decreased by 60 days (Fig. 4C). In contrast, PPAR-γ agonism markedly reorganized transcriptional dynamics in NCL-T. At early time points, pioglitazone modulated growth factor-related pathways (Fig. 4D), whereas at 30 days it induced coordinated downregulation of inflammatory, TGF-β–responsive, and ECM-organizational pathways (Fig. 4E). Consistent with the CL analysis, gene-level classification revealed a predominance of effective or incomplete normalization of fibrogenic transcripts in NCL-T (Fig. 4G), supporting a robust preventive transcriptional response. Upregulation of the TGF-β transcriptional repressor *Tgif1* further supported attenuation of TGF-β signaling (Fig. 4E, 4G). By 60 days, only a limited number of genes remained differentially expressed (Fig. 4F), consistent with resolution of the transcriptomic disturbance. Full transcriptional data, including fold change and adjusted p values, are provided in Supplementary Material 1.

To independently validate the transcriptomic findings, selected genes involved in inflammatory signaling and fibrogenesis were quantified by real-time qRT-PCR (Supplementary Material 1). This analysis confirmed that PPARγ treatment reduced the expression of key inflammatory mediators (*Nfkb1, Ccl3*) and fibrogenic markers (*Acta2, Col1a2, Lox, Tgfb3, Tgif1 and Mmp2*), corroborating the array-based findings and supporting the biological robustness of the observed transcriptional remodeling.

### Gene co-expression network analysis revealed distinct topology between groups

**Fig. 5:**
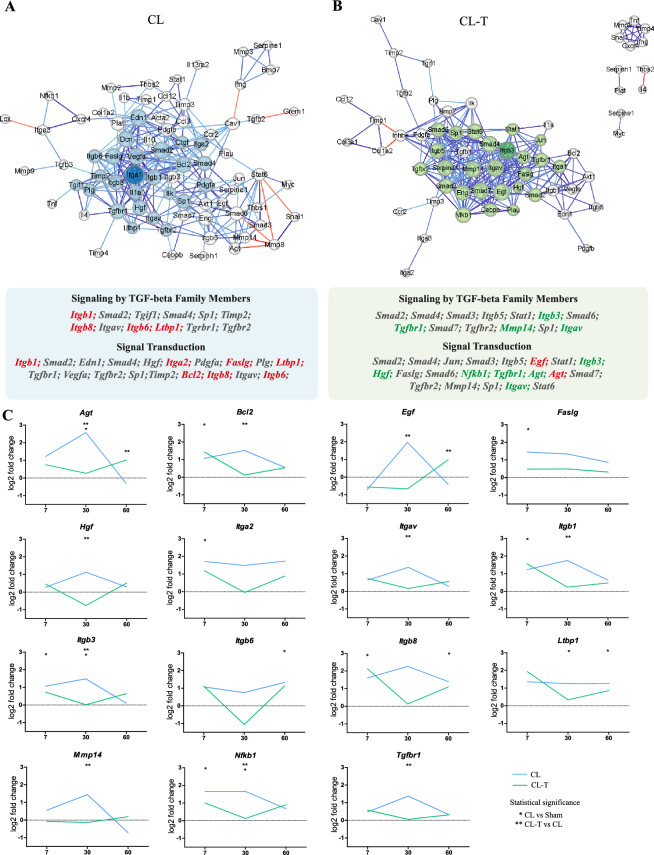
Gene co-expression network and gene expression changes in the cholestatic lobe following PPAR-γ agonist treatment. In the gene networks, hub genes are shown in darker shades and their first neighbors in lighter shades. (**A**) Vehicle-treated cholestatic lobe (CL) exhibits a disorganized topology, i.e., with high connectivity and absence of clear hierarchical or modular structure. The hub gene *Itga1* and the first neighbor genes were subjected to Reactome pathway enrichment analysis (blue box). (**B**) PPAR-γ agonist-treated cholestic lobe (CL-T) similarly exhibits a disorganized network topology, indicating that pharmacological intervention does not restore hierarchical organization. The hub gene *Itgb3* and the first neighbor genes were analyzed by Reactome pathway enrichment (green box). Node color indicates gene regulation status: red, upregulated; green, downregulated; grey, not differentially expressed. (**C**) Expression profiles of differentially expressed genes from selected modules at 7, 30, and 60 days post-surgery, expressed as log2 fold change relative to sham. CL, vehicle-treated; CL-T, PPAR-γ agonist–treated. Statistical analysis was performed using Student’s t-test with Benjamini–Hochberg correction. Asterisks indicate statistically significant differences: *CL vs sham; **CL-T vs CL.

**Fig. 6:**
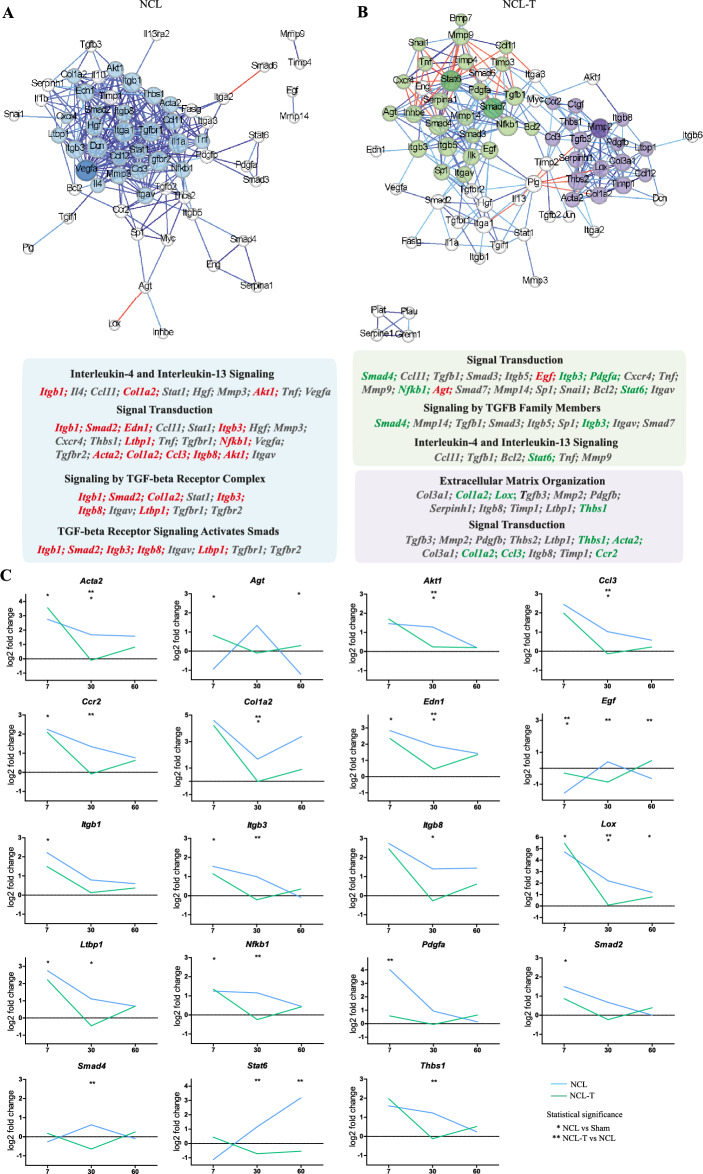
Gene co-expression network and gene expression changes in the non-cholestatic lobe following PPAR-γ agonist treatment. In the gene networks, hub genes are shown in darker shades and their first neighbors in lighter shades. (**A**) Vehicle-treated non-cholestatic lobe (NCL) exhibits a disorganized topology, i.e., presented high connectivity and lack of clear hierarchical structure. The hub gene *Vegfa* and the first neighbor genes were subjected to Reactome pathway enrichment analysis (blue box). (**B**) In contrast, the PPAR-γ agonist-treated non-cholestatic lobe (NCL-T) displayed a more organized network topology with two modules. Genes from each module were subjected to Reactome pathway enrichment analysis (green and purple boxes). Node color indicates gene regulation status: red, upregulated; green, downregulated; grey, not differentially expressed. (**C**) Expression profiles of differentially expressed genes from selected modules at 7, 30, and 60 days post-surgery, expressed as log2 fold change relative to sham. NCL, vehicle-treated; NCL-T, PPAR-γ agonist–treated. Statistical analysis was performed using Student’s t-test with Benjamini–Hochberg correction. Asterisks indicate statistically significant differences: *NCL vs sham; **NCL-T vs NCL.

Gene co-expression network analysis demonstrated that both the vehicle-treated CL and NCL networks exhibited dense but poorly modular architectures, characterized by diffuse connectivity, weak gene-gene correlations, absence of well-defined functional clusters, and lack of hierarchical organization (Fig. 5A and 6A). This disordered topology is consistent with broad transcriptional dysregulation probably associated with sustained fibro-inflammatory signaling during segmental cholestasis. In vehicle-treated CL, the network was organized around the hub gene *Itga1*. Functional enrichment analysis of the *Itga1* and its first-neighbor genes revealed enrichment of pathways related to signaling by TGF-beta family members and signal transduction, with predominant upregulation of genes *Bcl2, Faslg, Itga2, Itgb1, Itgb6, Itgb8* and *Ltbp* (Fig. 5C). These results reflect a fibrogenic and adhesion-related transcriptional core. Similarly, the vehicle-treated NCL network was centered on *Vegfa*. Functional analysis of *Vegfa* and its first-neighbor genes revealed enrichment of pathways related to interleukin-4 and interleukin-13 signaling, signal transduction, TGF-beta receptor complex signaling and TGF-beta receptor signaling activates Smads, with upregulation of genes *Acta2, Akt1, Ccl3, Col1a2, Edn1, Itgb1, Itgb3, Itgb8, Ltbp1, Nfkb1* and *Smad2* (Fig. 6C).

In CL-T, PPAR-γ agonism markedly attenuated inflammatory and fibrogenic transcriptional pathways, as reflected by reduced expression of key genes involved in extracellular matrix remodeling, cytokine signaling, and TGF-β-related pathways. However, this molecular attenuation did not translate into restoration of higher-order network organization. The CL-T network remained poorly modular, with low connectivity and limited functional clustering, indicating that pioglitazone suppresses pathway activation but has minimal impact on the higher-order organization of gene–gene relationships in tissue subjected to persistent obstructive injury (Fig. 5B; 5C). Notably, the CL-T network was organized around the hub gene *Itgb3*, suggesting a shift toward integrin-mediated signaling without recovery of coordinated regulatory architecture. Detailed Reactome enrichment results for network-associated genes are provided in Supplementary Material 2.

In contrast, the most pronounced network-level effects of PPAR-γ agonism were observed in the non-cholestatic lobe (Fig.6). While the vehicle-treated NCL network resembled the disorganized profiles of CL and CL-T, being marked by low modularity, weak connectivity, and absence of hierarchical structure (Fig. 6A), PPAR-γ treatment induced extensive transcriptional reorganization in NCL-T. The NCL-T network exhibited clearly defined modules, coordinated positive and negative interactions, and regulatory hubs linking distinct functional clusters, including pathways related to signal transduction, extracellular matrix organization, and cytokine-mediated signaling (Fig. 6B). Notably, module 1 was organized around *Stat6* and *Smad7* as central hubs, consistent with coordinated anti-inflammatory and anti-fibrotic regulatory signaling, whereas module 2 was centered on *Mmp2*, indicating controlled extracellular matrix remodeling. Reactome pathway enrichment analysis for each module is detailed in Supplementary Material 2. This distinction reinforces that transcriptional changes observed in CL-T reflect partial pathway suppression without restoration of regulatory organization, in contrast to the coordinated normalization observed in NCL-T, highlighting the greater transcriptional plasticity of the lobe not subjected to chronic fibrogenic drive (Fig. 6C).

## Discussion

In this study, we demonstrate that PPARγ agonism exerts a region-dependent antifibrotic effect during segmental cholestasis, preserving transcriptional organization and limiting fibro-inflammatory propagation in non-cholestatic liver regions, while established cholestatic niches remain largely refractory to modulation. Using a selective bile duct ligation (sBDL) model that reproduces key features of segmental biliary obstruction,^21–23^ we provide mechanistic insight into how inflammatory and fibrogenic networks evolve differentially within distinct hepatic compartments exposed to heterogeneous injury. Importantly, this experimental approach enables the simultaneous evaluation of fibrogenic responses in anatomically adjacent yet functionally distinct hepatic regions within the same organism. This approach highlights the spatial heterogeneity of the injury response and provides a unique opportunity to investigate how the local microenvironment modulates antifibrotic responsiveness, a feature that cannot be adequately addressed in conventional total bile duct ligation models.

Segmental cholestasis represents a clinically relevant but underexplored condition, particularly in pediatric settings such as post-Kasai biliary atresia and post-transplant biliary strictures, where fibrosis often progresses despite partial preservation of bile flow.^2,17–20^ In contrast to total bile duct ligation (BDL), which induces diffuse and rapidly progressive liver injury, the sBDL model enables the investigation of segmental fibrogenic responses and their propagation into adjacent non-cholestatic regions.^44^ This experimental model therefore offers unique translational value for assessing antifibrotic strategies in contexts where fibrogenic stimuli differ substantially between hepatic compartments distant from the primary injury.^21–23^

PPARγ is expressed in various hepatic cell populations, including hepatocytes, cholangiocytes, hepatic stellate cells (HSCs), portal fibroblasts, and immune cells, and functions as a negative regulator of inflammatory signaling and myofibroblastic transdifferentiation.^21^ In the present model, pioglitazone attenuated inflammation and fibrosis induced by segmental cholestasis, particularly in the non-cholestatic lobe, suggesting that PPARγ activation modulates not only local inflammatory responses but also autocrine and paracrine signaling mechanisms that sustain fibrogenic progression beyond the primary site of injury.

Consistent with previous studies^45–50^ showing that PPARγ agonists suppress inflammatory responses initiated by TLR activation across hepatic cell types, network-based transcriptomic analysis suggested an association between pioglitazone treatment and reorganization of gene interaction patterns governing immune activation and fibrogenesis, rather than inducing uniform transcriptional suppression. In the non-cholestatic lobe of PPAR-γ agonist-treated animals (NCL-T), *Stat6* and *Smad7* emerged as central hubs within a hierarchically organized regulatory module, reflecting their shared role in coordinating inflammatory and fibrogenic signaling.

Pathway enrichment highlighted TGF-β receptor signaling, extracellular matrix organization, and signal transduction processes, with coordinated downregulation of key profibrotic and inflammatory genes, alongside preservation of trophic and regenerative signals. Notably, this transcriptional reorganization occurred without global gene silencing, supporting a network-level regulatory effect. Consistent with the gene-level classification, this pattern is best interpreted as effective or incomplete normalization of fibrogenic and inflammatory transcripts, rather than full transcriptional reversion to a quiescent state. This distinction reinforces the concept that PPARγ agonism primarily restores regulatory balance within pathogenic gene networks instead of inducing complete transcriptional normalization.

Importantly, the presence of two topologically distinct network modules in NCL-T indicates that PPARγ agonism appeared to preserve the hierarchical dysregulation characteristic of progressive fibrogenesis observed in vehicle-treated animals in NCL. The STAT6/Smad7-centered module encompassed upstream inflammatory and profibrogenic regulators associated with TGF-β and IL-4/IL-13 signaling, whereas a second module centered on MMP2 aggregated downstream extracellular matrix remodeling components and signal transduction pathways. This modular reorganization is consistent with transcriptional transrepression mechanisms mediated by PPARγ, including interference with NF-κB and STAT signaling, modulation of kinases activity, and competition for nuclear co-regulators.^50–54^ In accordance with this interpretation, STAT6 exhibited predominantly positive correlations with profibrotic genes and negative correlations with inflammatory mediators, such as TNF and CXCR4, as well as with CCL11, a chemokine associated with Th2-driven immune responses. These network relationships are consistent with modulation of the IL-4/IL-13–STAT6–TGF-β signaling axis, a pathway known to influence macrophage polarization, myofibroblast activation, and matrix deposition in NCL-T. This observation is consistent with the immunomodulatory role of PPARγ signaling and supports the concept that PPARγ activation may exert a preventive antifibrotic effect in obstructive cholestasis by modulating fibro-inflammatory signaling before fibrogenic remodeling becomes fully established.

In parallel with these transcriptional changes, PPARγ agonism also modulated cellular turnover in the non-cholestatic lobe. The early increase in hepatocyte apoptosis observed in NCL-T, accompanied by a robust Ki-67–positive proliferative response, suggests that PPARγ activation promotes controlled cellular turnover rather than progressive tissue injury. This transient apoptotic signal, confined to early time points, likely reflects the elimination of damaged hepatocytes followed by compensatory regeneration, contributing to restoration of tissue homeostasis. The persistence of apoptotic signaling in CL-T, however, underscores the dominant impact of sustained biliary obstruction, which constrains regenerative capacity despite pharmacological intervention.

In contrast, vehicle-treated NCL exhibited enrichment of IL-4/IL-13 signaling and a dysregulated co-expression network centered on VEGFA, positively connected to TGF-β signaling components and integrin-mediated signal transduction. Notably, the absence of clearly defined modules reflects a loss of hierarchical organization, favoring uncontrolled crosstalk among inflammatory, angiogenic, and fibrogenic pathways. This transcriptional architecture correlated with the observed phenotype, characterized by accumulation of CD68⁺ macrophages, pronounced ductular reaction, and periportal hepatocellular injury, consistent with a classical fibro-inflammatory microenvironment.

Recent evidence highlights the role of cholangiocyte-intrinsic inflammatory signaling in ductular expansion and fibrosis progression in cholestatic models.^8,55,56^ Activation of the non-canonical NF-κB pathway via NF-κB-inducing kinase (NIK) promotes cholangiocyte proliferation, resistance to apoptosis, and secretion of cholangiokines by reactive cholangiocytes.^55^ These reactive cholangiocytes also exhibit strong TLR4-dependent inflammatory responses, characterized by sustained NF-κB activation and production of cytokines and growth factors.^57–59^ PPARγ activation has been shown to repress NF-κB–dependent inflammation in biliary epithelium through upregulation of IκBα,^60^ providing a mechanistic basis for our observation that pioglitazone reduced ductular proliferation and cholangiocyte activation in NCL-T. Thus, suppression of STAT6-centered inflammatory networks may have contributed to the attenuation of fibrogenic progression in regions secondary to injury.

Collectively, PPARγ agonism in NCL-T was associated with attenuation of the transition from inflammatory signaling to activation of fibrogenic pathways, suppressing genes involved in TGF-β signaling, ECM organization, and IL-4/IL-13 pathways. These effects likely reflect both direct transcriptional repression by PPARγ and indirect consequences of reduced inflammatory crosstalk sustaining fibrogenic cell activation. These findings reinforce previous evidence showing that PPARγ agonists reduce hepatic fibrosis by modulating pro-fibrogenic gene expression, limiting expansion of α-SMA⁺ HSCs, and preventing excessive ECM accumulation.^31,61^ Nonetheless, the antifibrotic efficacy of PPARγ appears to depend on the timing of therapeutic intervention and the extent of tissue injury.^30–32^ Notably, Leclercq et al. demonstrated that, whereas CCl₄-induced fibrosis responds to PPARγ modulation, fibrosis resulting from BDL remains refractory even when pioglitazone is administered early, suggesting that the persistence and severity of cholestatic injury may exceed the modulatory capacity of this receptor.^30^ These observations further support a predominantly preventive rather than curative role for PPARγ agonism in fibrogenic liver disease.

Indeed, PPARγ agonism in the cholestatic lobe (CL-T) showed limited efficacy, revealing a microenvironment refractory to antifibrotic modulation despite prolonged treatment. Early molecular events had already established a consolidated fibro-inflammatory network enriched for TGF-β signaling and signal transduction pathways, accompanied by persistent macrophage infiltration, ductular proliferation, myofibroblast activation, and collagen deposition. These features resemble classical BDL models, in which the severity and persistence of bile acid-mediated injury exceed the modulatory capacity of PPARγ.

One plausible explanation involves sustained accumulation of toxic bile acids, leading to hyperactivation of nuclear receptors such as FXR, TGR5, and PXR, which compete with PPARγ for shared nuclear co-regulators and limit its transrepressive capacity.^62–66^ Simultaneously, sustained activation of TLR4, NIK, and NF-κB, widely documented in cholangiopathies, reinforces inflammatory and mitogenic signaling, continuously driving HSC activation and matrix deposition.^55,57,67,68^ Although late-stage transcriptional repression of selected inflammatory and fibrotigenic genes was observed, these changes were insufficient to redirect the disease trajectory once a consolidated fibrotic niche had been established. Consistent with this interpretation, the late downregulation of structural matrix and remodeling-related genes observed in CL-T at 60 days likely reflects attenuation of active fibrogenic turnover without histological reversal, rather than true fibrosis regression. Accordingly, this pattern is best classified as incomplete normalization, characterized by transcriptional attenuation without phenotypic reversal of established fibrosis. This transcriptional shift suggests a transition toward matrix stabilization in tissue exposed to persistent obstructive injury, in agreement with the absence of histological improvement and the lack of restored network modularity in CL-T.

Analysis of tissue remodeling pathways in the CL-T revealed coordinated downregulation of TGF-β–related signaling components and extracellular matrix–associated genes, alongside reduced expression of signal transduction mediators. Additionally, simultaneous repression of both plasminogen activator (*Plau*-uPA) and its inhibitor *Serpine,* suggests a reorganization of extracellular matrix turnover dynamics. However, this transcriptional modulation did not translate into architectural remodeling, indicative of a stabilized fibrotic state in which both matrix deposition and degradation are concurrently reduced. Suppression of inflammatory transcription factors such as NF-κB and AP-1 may secondarily limit induction of fibrillar matrix metalloproteinases and uPA-dependent plasmin generation, thereby constraining effective matrix degradation.^69–75^ Consequently, highly cross-linked collagen matrices characteristic of advanced fibrosis remains largely resistant to PPARγ-mediated modulation.

In line with this interpretation, downregulation of angiogenic mediators such as *Vegfa* in CL-T, suggesting attenuation of pathological microvascular remodeling, an effect previously associated with PPAR modulation.^76^ However, while pioglitazone effectively dampened inflammatory and angiogenic signaling, these effects were insufficient to induce regression of established fibrosis in CL-T, highlighting that repression of inflammatory signaling alone is insufficient to trigger active fibrosis resolution. This is biologically consistent, as PPARγ activation attenuates the transcription of pro-inflammatory and pro-fibrogenic pathways but does not directly engage pro-resolution mechanisms required for matrix degradation. Moreover, suppression of NF-κB and AP-1 may secondarily limit the induction of fibrillar MMPs and uPA-dependent plasmin generation, which are essential for ECM degradation.^77–79^ Consequently, established fibrosis, characterized by highly cross-linked collagen stabilized by LOX/LOXL, remains largely refractory to PPARγ agonism. In contrast, in the non-cholestatic lobe PPAR-γ agonist-treated (NCL-T) where injury was secondary and fibrosis less advanced, PPARγ activation preserved parenchymal architecture, reduced fibro-inflammatory signaling, and prevented fibrotic progression, highlighting a predominantly preventive rather than curative role for PPARγ agonism in segmental cholestasis.

These findings align with clinical evidence demonstrating that PPARγ agonists attenuate hepatic inflammation and, under specific conditions, improve fibrosis outcomes, as observed in nonalcoholic steatohepatitis (NASH).^80–82^ Despite the etiopathogenic differences between NASH and cholestatic fibrosis, both share central fibrogenic pathways, including TGF-β1, α-SMA, and PPAR modulation^83^. Nevertheless, extrapolation to cholestatic diseases requires caution, as the pathophysiology of cholestasis, especially in segmental phenotypes, involves unique patterns of inflammation and extracellular matrix dynamics. Our study addresses a critical knowledge gap by demonstrating that preservation of bile flow and injury heterogeneity are key determinants of therapeutic responsiveness. PPARγ activation effectively limits fibrogenic propagation into non-cholestatic regions but is insufficient to reverse fibrosis once a cholestatic microenvironment becomes fully consolidated. These findings redefine the understanding of the role of PPARγ in cholestatic fibrogenesis and highlight the scarcity of studies addressing these mechanisms in pediatric contexts.

Finally, we acknowledge as a limitation that treatment initiation occurred after early fibrogenic events had already been established, which may have reduced therapeutic responsiveness in the cholestatic lobe. This design choice was intentional and clinically relevant, reflecting clinical scenarios in which patients with biliary strictures often present with established fibrosis at diagnosis. From this perspective, our findings provide valuable insight into the therapeutic window during which PPARγ modulation may exert maximal benefit. Another limitation of our study is that tissue concentrations of pioglitazone were not directly measured. Therefore, we cannot formally exclude the possibility that altered drug distribution within the obstructed hepatic lobe may have contributed to the limited therapeutic response observed in CL-T. However, the histological and transcriptional findings obtained in our model suggest that the reduced responsiveness of the cholestatic lobe is more likely related to the establishment of a stabilized fibro-inflammatory microenvironment rather than exclusively to impaired drug bioavailability. In addition, our transcriptional analysis focused primarily on inflammatory and fibrogenic pathways, and genes involved in bile acid transport and FXR signaling were not included in the PCR array panels used. Future studies investigating the regulation of bile acid transporters and nuclear receptors involved in bile acid homeostasis, such as FXR, may provide additional insight into adaptive responses to localized cholestasis and their interaction with fibro-inflammatory signaling pathways.

In conclusion, this study demonstrates that PPARγ agonism was associated with a region-dependent antifibrotic effect during segmental cholestasis, preserving parenchymal organization and limiting fibrogenic progression in non-cholestatic liver regions. In contrast, fibrosis established within cholestatic lobes remains refractory to PPARγ modulation, reflecting a consolidated microenvironment whose reversal likely requires combinatorial therapeutic approaches capable of actively promoting extracellular matrix degradation. From a translational perspective, these findings help explain the clinical heterogeneity observed among patients with partial biliary obstruction and suggest that PPARγ activation may be most effective when applied early, preserving the remaining functional hepatic parenchyma before fibrotic remodeling becomes fully consolidated. Together, these observations provide mechanistic insight into how antifibrotic strategies targeting PPARγ signaling may help preserve functional hepatic parenchyma in clinical conditions characterized by segmental biliary injury. In this context, PPARγ activation may act by limiting the expansion of fibrogenic activation into liver regions with preserved biliary drainage, where tissue homeostasis remains sufficiently maintained to respond to antifibrotic modulation. This concept is particularly relevant for pediatric and post-transplant settings characterized by segmental cholestasis, in which fibrosis may continue to progress despite partial restoration of bile flow and where targeted antifibrotic therapies are currently lacking.

## Data availability 

The data supporting the findings of this study are available from the corresponding author upon reasonable request.

## Supplementary Information

Supplementary Information 1.

Supplementary Information 2.

## Abbreviations

α-SMA: Alpha-smooth muscle actin
ALT: Alanine aminotransferase
AP-1: Activator protein 1
AST: Aspartate aminotransferase
BDL: Bile duct ligation
BSA: Bovine serum albumin
CCl₄: Carbon tetrachloride
CL: Cholestatic lobe
CL-T: Cholestatic lobe treated with PPARγ agonist
CONCEA: Brazilian National Council for the Control of Animal Experimentation
Ct: Cycle threshold
CXCR4: C-X-C motif chemokine receptor 4
DAMPs: Damage-associated molecular patterns
ECM: Extracellular matrix
FXR: Farnesoid X receptor
GGT: Gamma-glutamyl transferase
H&E: Hematoxylin and eosin
HSCs: Hepatic stellate cells
IL: Interleukin
LOX/LOXL: Lysyl oxidase / Lysyl oxidase-like
MMP: Matrix metalloproteinase
NASH: Nonalcoholic steatohepatitis
NCL: Non-cholestatic lobe
NCL-T: Non-cholestatic lobe treated with PPARγ agonist
NF-κB: Nuclear factor kappa B
NIK: NF-κB–inducing kinase
**NTC**: No-template control
PCR: Polymerase chain reaction
POD: Postoperative day
PPARγ: Peroxisome proliferator–activated receptor gamma
PXR: Pregnane X receptor
qRT-PCR: Quantitative real-time polymerase chain reaction
sBDL: Selective bile duct ligation
STAT6: Signal transducer and activator of transcription 6
TGF-β: Transforming growth factor beta
TLR: Toll-like receptor
TGR5: Takeda G-protein–coupled receptor 5
uPA: Urokinase-type plasminogen activator
VEGFA: Vascular endothelial growth factor A
