# Supplementary material for: Residual bile flow shapes PPARγ-mediated antifibrotic responses in experimental segmental cholestasis
Source: Sci Rep. 2026 Apr 25;16:19160. doi: 10.1038/s41598-026-50023-1 (PMC13279945; doi:10.1038/s41598-026-50023-1)

### Residual bile flow shapes PPARγ-mediated antifibrotic responses in experimental segmental cholestasis

Josiane O Gonçalves,^1,2^ Cleiton A.M Mafra,^3^ Isac Castro,^4^ Bruno Cogliati,^5^ Silvia Y Bando,^2^ Walcy R. Teodoro, ^6^ Suellen Serafini,^1,2^ Lorenzo F Ferreira,^1,2^ Carlos A Moreira-Filho,^2^ Uenis Tannuri,^1,2^ Ana C.A Tannuri.^1,2^

^1^ Division of Pediatric Surgery, Pediatric Liver Transplant Unit, and Pediatric Surgery Research Laboratory (LIM 30), University of São Paulo School of Medicine, São Paulo, SP, Brazil.

^2^ Department of Pediatrics, University of São Paulo School of Medicine, São Paulo, SP, Brazil.
^3^ Liver and Gastrointestinal Transplant Division, Department of Gastroenterology, University of São Paulo School of Medicine, São Paulo, SP, Brazil.
^4^ Department of Nephrology, University of São Paulo School of Medicine, São Paulo, SP, Brazil.
^5^ Department of Pathology, School of Veterinary Medicine and Animal Science, University of São Paulo, São Paulo, SP, Brazil.
^6^ Division of Rheumatology, Hospital das Clínicas, University of São Paulo School of Medicine, São Paulo, SP, Brazil.

**Supplementary Table 1.** Stability ranking of candidate housekeeping genes in rat liver samples based on RefFinder analysis.

| **Gene** | **geNorm_M** | **NormFinder_SV** | **BestKeeper_SD** | **BestKeeper_CV%** | **Delta CT_rank** | **RefFinder_rank** | **Stability assessment** |
| --- | --- | --- | --- | --- | --- | --- | --- |
| *HPRT1* | 0.380 | 0.250 | 0.08 | 0.42 | 0.76 | 1.00 | Stable |
| *RPLP1* | 0.380 | 0.496 | 0.23 | 1.31 | 0.83 | 1.86 | Stable |
| *Actin-beta* | 0.723 | 0.447 | 0.53 | 3.83 | 0.89 | 3.13 | Moderately stable |
| *B2M* | 0.556 | 0.791 | 0.44 | 2.56 | 0.99 | 3.46 | Unstable |
| *YWHAZ* | 0.955 | 1.219 | 0.97 | 5.64 | 1.30 | 5.00 | Unstable |

**Supplementary Table 2.** Differentially expressed genes in the cholestatic lobe (CL) compared with sham-operated animals at 7, 30, and 60 days post-surgery (fold change ≥2.0 or ≤−2.0; p < 0.05).

| Days post-surgery | Gene symbol | Fold change | P value |
| --- | --- | --- | --- |
| 7 days | *Lox* | 27.31 | 0.001 |
|  | *Serpine1* | 19.99 | 0.010 |
|  | *Mmp9* | 12.65 | 0.005 |
|  | *Myc* | 8.55 | 0.031 |
|  | *Col1a2* | 8.08 | 0.005 |
|  | *Timp1* | 7.51 | 0.012 |
|  | *Dcn* | 6.26 | 0.005 |
|  | *Acta2* | 6.16 | 0.025 |
|  | *Thbs2* | 5.70 | 0.013 |
|  | *Tgfb3* | 5.02 | 0.026 |
|  | *Plat* | 4.85 | 0.005 |
|  | *Mmp2* | 4.24 | 0.005 |
|  | *Col3a1* | 3.76 | 0.004 |
|  | *Itga3* | 3.75 | 0.040 |
|  | *Serpinh1* | 3.65 | 0.0008 |
|  | *Itga2* | 3.27 | 0.021 |
|  | *Pdgfb* | 3.21 | 0.030 |
|  | *Edn1* | 3.19 | 0.0004 |
|  | *Nfkb1* | 3.16 | 0.0007 |
|  | *Itgb8* | 3.03 | 0.014 |
|  | *Ctgf* | 2.88 | 0.037 |
|  | *Stat6* | 2.75 | 0.007 |
|  | *Faslg* | 2.72 | 0.017 |
|  | *Ccr2* | 2.65 | 0.032 |
|  | *Cav1* | 2.62 | 0.016 |
|  | *Ltbp1* | 2.56 | 0.001 |
|  | *Plau* | 2.50 | 0.036 |
|  | *Akt1* | 2.46 | 0.001 |
|  | *Itgb1* | 2.35 | 0.001 |
|  | *Timp3* | 2.17 | 0.001 |
|  | *Bcl2* | 2.10 | 0.025 |
|  | *Itgb3* | 2.09 | 0.002 |
|  | *Inhbe* | -2.09 | 0.037 |
| 30 days | *Serpine1* | 24.02 | 0.037 |
|  | *Agt* | 5.96 | 0.039 |
|  | *Myc* | 5.69 | 0.048 |
|  | *Tgfb3* | 4.96 | 0.036 |
|  | *Akt1* | 4.44 | 0.045 |
|  | *Thbs1* | 3.93 | 0.034 |
|  | *Ctgf* | 3.92 | 0.006 |
|  | *Ccr2* | 3.43 | 0.001 |
|  | *Mmp2* | 3.30 | 0.026 |
|  | *Stat6* | 3.28 | 0.034 |
|  | *Nfkb1* | 3.15 | 0.033 |
|  | *Timp3* | 2.94 | 0.005 |
|  | *Timp1* | 2.86 | 0.033 |
|  | *Eng* | 2.84 | 0.039 |
|  | *Plau* | 2.83 | 0.045 |
|  | *Itgb3* | 2.79 | 0.026 |
|  | *Acta2* | 2.69 | 0.045 |
|  | *Smad6* | 2.54 | 0.012 |
|  | *Col1a2* | 2.42 | 0.045 |
|  | *Ltbp1* | 2.38 | 0.007 |
| 60 days | *Col1a2* | 4.62 | 0.029 |
|  | *Serpine1* | 3.87 | 0.018 |
|  | *Acta2* | 3.59 | 0.005 |
|  | *Itgb8* | 2.63 | 0.008 |
|  | *Itgb6* | 2.51 | 0.023 |
|  | *Ltbp1* | 2.40 | 0.024 |
|  | *Edn1* | 2.35 | 0.004 |
|  | *Plat* | 2.29 | 0.0007 |
|  | *Tgfb3* | 2.26 | 0.0000 |
|  | *Itga3* | 2.13 | 0.007 |
|  | *Ctgf* | 2.05 | 0.047 |
|  | *Inhbe* | -2.37 | 0.026 |

**Supplementary Table 3.** Differentially expressed genes in the cholestatic lobe of **PPARγ agonist–treated animals (CL-T)** compared with **vehicle-treated cholestatic lobe animals (CL)** at 7, 30, and 60 days post-surgery (fold change ≥2.0 or ≤−2.0; p < 0.05).

| Days post-surgery | Gene symbol | Fold change | P value |
| --- | --- | --- | --- |
| 7 days | - | *-* | - |
| 30 days | *Tgfb3* | -2.14 | 0.048 |
|  | *Timp1* | -2.16 | 0.013 |
|  | *Itgav* | -2.30 | 0.026 |
|  | *Itga1* | -2.41 | 0.041 |
|  | *Mmp2* | -2.49 | 0.019 |
|  | *Tgfbr1* | -2.49 | 0.041 |
|  | *Il1b* | -2.57 | 0.029 |
|  | *Bcl2* | -2.63 | 0.024 |
|  | *Itgb3* | -2.76 | 0.006 |
|  | *Pdgfa* | -2.81 | 0.026 |
|  | *Itgb1* | -2.84 | 0.041 |
|  | *Nfkb1* | -2.90 | 0.011 |
|  | *Mmp14* | -2.99 | 0.037 |
|  | *Eng* | -3.13 | 0.008 |
|  | *Tnf* | -3.15 | 0.048 |
|  | *Akt1* | -3.18 | 0.018 |
|  | *Thbs1* | -3.36 | 0.009 |
|  | *Myc* | -3.48 | 0.022 |
|  | *Hgf* | -3.68 | 0.006 |
|  | *Ctgf* | -3.74 | 0.001 |
|  | *Ccl3* | -3.77 | 0.014 |
|  | *Timp3* | -3.94 | 0.025 |
|  | *Plau* | -4.38 | 0.009 |
|  | *Il10* | -4.61 | 0.024 |
|  | *Agt* | -4.97 | 0.015 |
|  | *Egf* | -6.23 | 0.028 |
|  | *Vegfa* | -6.91 | 0.048 |
|  | *Serpine1* | -9.93 | 0.010 |
| 60 days | *Egf* | 2.63 | 0.004 |
|  | *Agt* | 2.49 | 0.004 |
|  | *Cxcr4* | 2.14 | 0.019 |
|  | *Col1a2* | -2.45 | 0.015 |
|  | *Mmp1* | -4.43 | 0.041 |

**Supplementary Table 4**. Differentially expressed genes in the non-cholestatic lobe (NCL) compared with sham-operated animals at 7, 30, and 60 days post-surgery (fold change ≥2.0 or ≤−2.0; p < 0.05).

| Days post-surgery | Gene symbol | *Fold change* | P value |
| --- | --- | --- | --- |
| 7 days | *Lox* | 26.37 | 0.013 |
|  | *Timp1* | 22.13 | 0.007 |
|  | *Dcn* | 19.57 | 0.033 |
|  | *Serpine1* | 15.60 | 0.037 |
|  | *Grem1* | 14.15 | 0.022 |
|  | *Mmp13* | 10.90 | 0.028 |
|  | *Tgfb3* | 7.91 | 0.041 |
|  | *Edn1* | 7.15 | 0.021 |
|  | *Acta2* | 6.77 | 0.012 |
|  | *Ltbp1* | 6.73 | 0.021 |
|  | *Col3a1* | 6.09 | 0.021 |
|  | *Ccr2* | 4.74 | 0.012 |
|  | *Itgb1* | 4.65 | 0.046 |
|  | *Il13ra2* | 4.15 | 0.028 |
|  | *Mmp2* | 3.65 | 0.017 |
|  | *Serpinh1* | 3.26 | 0.010 |
|  | *Ctgf* | 3.23 | 0.018 |
|  | *Bcl2* | 2.96 | 0.015 |
|  | *Faslg* | 2.96 | 0.025 |
|  | *Itgb3* | 2.90 | 0.023 |
|  | *Smad2* | 2.82 | 0.029 |
|  | *Nfkb1* | 2.37 | 0.037 |
|  | *Egf* | -2.97 | 0.003 |
|  | *Inhbe* | -16.90 | 0.004 |
| 30 days | *Mmp8* | 8.01 | 0.018 |
|  | *Lox* | 4.59 | 0.010 |
|  | *Edn1* | 3.73 | 0.024 |
|  | *Tgfb3* | 3.43 | 0.017 |
|  | *Acta2* | 3.20 | 0.006 |
|  | *Col1a2* | 3.20 | 0.022 |
|  | *Itgb6* | 3.00 | 0.034 |
|  | *Ctgf* | 2.70 | 0.036 |
|  | *Itgb8* | 2.64 | 0.023 |
|  | *Akt1* | 2.42 | 0.046 |
|  | *Ltbp1* | 2.16 | 0.039 |
|  | *Ccl3* | 2.03 | 0.021 |
| 60 days | *Lox* | 2.29 | 0.020 |

**Supplementary Table 5.** Differentially expressed genes in the **non-cholestatic lobe** of **PPARγ agonist–treated animals (NCL-T)** compared with **vehicle-treated non-cholestatic lobe animals (NCL)** at 7, 30, and 60 days post-surgery (fold change ≥2.0 or ≤−2.0; p < 0.05).

| Days post-surgery | Gene symbol | *Fold change* | P value |
| --- | --- | --- | --- |
| 7 days | *Agt* | 3.39 | 0.020 |
|  | *Egf* | 2.41 | 0.031 |
|  | *Pdgfa* | -10.89 | 0.038 |
| 30 days | *Tgif1* | 2.06 | 0.005 |
|  | *Akt1* | -2.04 | 0.019 |
|  | *Tgfbr2* | -2.13 | 0.005 |
|  | *Eng* | -2.17 | 0.034 |
|  | *Vegfa* | -2.21 | 0.008 |
|  | *Ccl3* | -2.23 | 0.004 |
|  | *Jun* | -2.26 | 0.026 |
|  | *Itgb3* | -2.30 | 0.020 |
|  | *Smad6* | -2.33 | 0.044 |
|  | *Acta2* | -2.34 | 0.011 |
|  | *Egf* | -2.38 | 0.037 |
|  | *Smad4* | -2.40 | 0.004 |
|  | *Timp3* | -2.41 | 0.014 |
|  | *Thbs1* | -2.55 | 0.021 |
|  | *Il1b* | -2.58 | 0.030 |
|  | *Nfkb1* | -2.65 | 0.034 |
|  | *Edn1* | -2.70 | 0.010 |
|  | *Ccr2* | -2.71 | 0.037 |
|  | *Itgb6* | -2.79 | 0.011 |
|  | *Col1a2* | -3.22 | 0.006 |
|  | *Stat6* | -3.67 | 0.049 |
|  | *Lox* | -4.33 | 0.008 |
|  | *Mmp8* | -9.31 | 0.003 |
| 60 days | *Jun* | 3.13 | 0.005 |
|  | *Agt* | 2.84 | 0.018 |
|  | *Egf* | 2.18 | <0.001 |

**Supplementary Figure S1. Supplementary Figure S1. Validation of RT-PCR array results by quantitative real-time PCR (qRT-PCR) for selected genes in rat liver samples.** Dot plots show median relative expression levels calculated using ΔCt values. (A) Acta2; (B) Ccl3; (C) Col1a2; (D) Lox; (E) Mmp2; (F) Nfkb1; (G) Stat6; (H) Tgfb3; and (I) Tgif1.


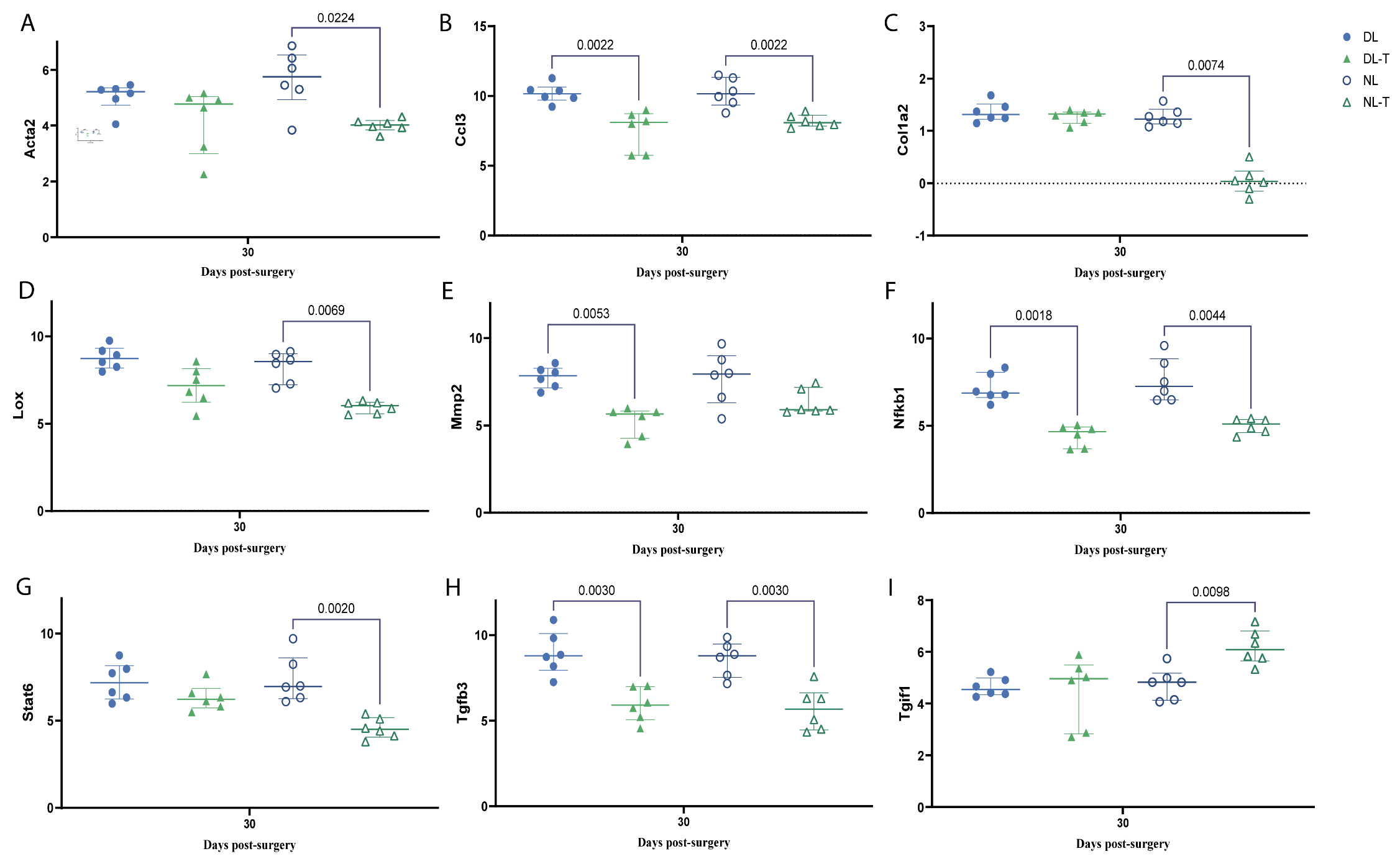

Supplement: Supplementary file 1 — Supplementary Information 1. [file 41598_2026_50023_MOESM1_ESM.docx]
